# Color characteristics of various computerized machinable ceramics veneered to yttria-stabilized tetragonal zirconia polycrystalline upon different hybridized techniques

**DOI:** 10.4317/jced.62187

**Published:** 2025-01-01

**Authors:** Niwut Juntavee, Apa Juntavee, Siripim Tangsatchatham

**Affiliations:** 1 Department of Prosthodontics, Faculty of Dentistry, Khon Kaen University, Khon Kaen, Thailand; 2 Division of Pediatric Dentistry, Department of Preventive Dentistry, Faculty of Dentistry, Khon Kaen University, Khon Kaen, Thailand; 3 Division of Biomaterials and Prosthodontics, Faculty of Dentistry, Khon Kaen University, Khon Kaen, Thailand

## Abstract

**Background:**

Hybridization technique impacted color of ceramic veneered zirconia. This study examined color characteristics of different ceramics veneered zirconia upon different hybridized techniques.

**Material and Methods:**

120 zirconia specimens (0.8 mm thickness, 12 mm diameter) were prepared from 3-yttria-stabilized tetragonal zirconia polycrystalline and unintentionally veneered with Vitabloc Mark-II (Vm), IPS e.max CAD (Em), Vita-Suprinity (Vs), and Celtra-Duo (Cd), by CAD-bonded (Cb) versus CAD-fused (Cf) hybridization (n=15/group). CIE-L*a*b* color characteristics were determined for translucency parameter (TP), contrast ratio (CR), opalescence parameter (OP), and color difference (ΔEdiff). Microstructures were investigated with SEM and XRD. Analysis of Variance and Bonferroni comparisons were determined for significant differences (*p*<0.05).

**Results:**

TP and OP were significantly higher, but lower CR and ΔEdiff for Vm and Em than Cd and VS. Cf hybridized technique significantly decreased TP and OP but increased CR and ΔEdiff than Cb, which amplified color alteration. Better TP and OP, with less CR and ΔEdiff, were achieved for zirconia veneering with either Vm or Em, compared to Vs or Cd, whether hybridized with Cb or Cf technique.

**Conclusions:**

Different veneering ceramics and hybridized techniques significantly altered color characteristics of ceramic veneered zirconia. Zirconia veneering with either Vm or Em appeared to produce better translucence and opalescence, with less contrast and color alteration than veneering with either Vs or Cd. CAD-fused decreased translucency, opalescence, and intensified color alteration due to t→m transformation. Nevertheless, the color alteration of ceramics veneered zirconia still rendered an acceptable limit, except for both Vs and Cd upon Cf hybridization.

** Key words:**CAD/CAM, color, contrast, hybridized technique, opalescence, translucency.

## Introduction

The progression in the technological elaboration of computer-aided design-computer-aided manufacturing (CAD/CAM) has become an increasingly useful technique for contemporary practice in dentistry (1). The evolution of this technology and the increasing demand for esthetics in dentistry commenced the dental practitioner and dental researcher to search for new ceramic materials that can render high-quality and reliable esthetic reconstruction (2). Amongst recently progressed ceramic materials, stabilized zirconia has gained increasing attractiveness as an exceptional replacement to metal owing to its auspicious aesthetics, biological compatibility, insignificant bacterial plaque accumulation together with optimal strength, and prime fracture toughness (3). Zirconia encompassed three microstructural phases: monoclinic (m-), tetragonal (t-), and cubic (c-) phases. These phases are interchangeable due to the triggered temperature. At the ambient temperature, the m-phase was detecTable. Upon heating up to 1,170ºC, the m-phase was induced to transform to the t-phase until the temperature reached 2,370ºC, and the c-phase appeared and remained unchanged up to the melting point of 2,680ºC. As cooling down, all the microstructures turn into the m-phase (4). To achieve and stabilize the desired t-phases at room temperature, a yttrium oxide stabilizer (Y2O3) was added. The 3 mol% Y2O3 was implemented into zirconia as called 3 mol% yttria-stabilized tetragonal polycrystalline (3Y-TZP) and presented almost the entire t-phase at the normal temperature. The superior advantages of 3Y-TZP restoration to endure the force of mastication founded on the metaphysical phase transitions from the t→m-phase as induced by external stimuli such as moisture, stress, and warmth, causing 4–5% volumetric expansion that enables stabilized zirconia as an exceptional strength called “transformation toughening” to prevent crack propagation and enhance strength for extensive restoration (4). However, zirconia is noticeably opaque white with a relatively high refractive index and diminishes light transmission (5,6). It essentially needs veneering ceramic to ensure aesthetic outcomes (7,8). The mechanical and optical properties allowed 3Y-TZP to be used as a substructure for veneering with translucent ceramic to render aesthetic restorations (5-7,9).

Traditional ceramic layering techniques have been used as the most typical technique in ceramic veneering zirconia. Several reports stated that ceramic veneered zirconia restorations predominantly deteriorated from cracking and delamination of the veneering ceramic up to 15-36% in 5 years of the follow-up period, which is the most commonly reported clinical complication (4,10). Although an exclusive prominent monolithic translucent 3Y-TZP has been introduced for the construction of absolute solid restorations to avoid chipping and delamination of veneering ceramic, its’ translucency still does not reach the desirable achievement (9). Hence, this circumstance restricts its usage as full-contoured restoration barely in the posterior region of the arch, yet it is still primarily used as a substructure for veneering with feldspathic or glass-ceramic (3). Alteration of the firing protocol has resulted in greater resistance to fracture of the ceramic veneered zirconia restorations (11). Other approaches have been established to increase the clinical implementation of ceramic veneered zirconia including the pressed-on ceramic veneering zirconia which is a technique for veneering zirconia by pressing procedure (12). However, the clinical outcome is still not fully satisfied and technique sensitive. The most recent strategy includes that both zirconia substructure and veneering ceramic are CAD/CAM generated which results in highly predicTable outcomes since the ceramic blanks are industrially produced with improved reliability and quality control (13). Several modern ceramics for CAD/CAM were introduced for this technique. For instance, lithium disilicate (LS2) glass-ceramic was proposed to enhance the mechanical property of feldspathic ceramic and it has been recommended as an alternative veneering ceramic for zirconia (13). More recently, zirconia-reinforced lithium silicate (ZLS) has been introduced as a new ceramic material proposed for CAD/CAM restoration. This syndicates the optimistic mechanical advantages of the zirconia with the esthetic appearance of glass ceramic, providing higher mechanical properties compared to LS2 glass ceramic (2,14). After the zirconia substructure and the veneering ceramic were CAD/CAM generated, both components were hybridized together by fusing with low fusion glass under appropriate sintering temperature (CAD-fused, Cf) or by bonding with resin cement (CAD-bonded, Cb). The Cf hybridized technique can produce a homogeneous multilayered restoration without initiating flaws or defective structures. While the Cb hybridized technique is more appropriate for an extensive reconstruction that does not need to be sintered, thereby, avoiding distortion from the sintering process (13).

The esthetic outcomes of ceramic veneered zirconia are influenced by types of veneering ceramic and their hybridized techniques (15,16). Veneering ceramic can enhance esthetic restoration by improving color characteristics of restorations in terms of translucency, contrast, opalescence, and color predictability (17,18). Translucence was defined by the quantity of light transmission across a material, which was identified as an existing condition between total opacity and transparency, and can be signified by the translucency parameter (TP) as well as contrast ratio (CR) (6,19). The material with superior translucence would permit greater light transmission and exhibited a greater TP but lesser CR values because both parameters are adversely associated. The crystalline structures, grain sizes, colorant mixtures, and porosities were stated to disturb the trajectory of light (20). The restoration should look blueish once the light is reflected out of it and emerge an orange manifestation once the light diffuses through the material. This occurrence is recognized as “opalescence”, signified by the opalescence parameter (OP), and attentively simulated appearance of human enamel (21,22). The supreme concern for dentists in providing aesthetic restoration for the patient is how to get the restoration fabricated by a dental technician with predicTable color as prescribed. Likewise, the dental technician is often frustrated with how to fabricate the restoration based on the material and existing technique to meet the dentist’s demand. Thus, different types of materials and techniques used for fabrication should produce the color appearance of restoration within an acceptable color perception (23). The color difference (∆Ediff) was employed to verify the level of perception, which was determined by the perceptibility threshold (PT, ∆Ediff = 2.6) and acceptability threshold (AT, ∆Ediff = 5.5). It indicated “clinically indistinguishable” as ∆Ediff ≤ 2.6, “clinically accepTable” as ∆Ediff = 2.6–5.5, and “clinically unaccepTable” as ∆Ediff > 5.5 (24).

Whilst computerized machinable ceramics veneered to zirconia upon hybridized techniques is a fairly new method, a lack of evidence regarding the color characteristics upon the hybridized techniques of contemporary machinable ceramics veneered zirconia substructure. Currently, LS2 ceramics is the only material intended to be connected with a zirconia substructure using fusion glass, other CAD/CAM-manufactured glass ceramics have never been used with this method (7,13). Moreover, previous studies commonly determined color perception by human judgment which restricted the capability to distinguish minute color differences and seem subjectively compared (1,6,10). To avoid non-reproducibility, color determination should be performed with a quantitative spectrophotometer (1). As such, the objective of this study was to evaluate the effect of different veneering ceramics and hybridized techniques on color characteristics of CAD/CAM fabricated ceramic veneered zirconia, including translucency, contrast, opalescence, and color alteration. The null hypothesis was that there was no significant difference in TP, CR, OP, and ∆Ediff of CAD/CAM generated ceramic veneering zirconia upon using different ceramics veneering, hybridization techniques, and these interactions.

## Material and Methods

**Figure 1:**
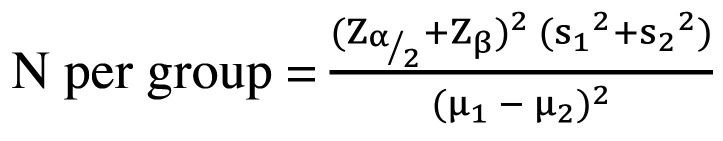
Equation 1.

This experimental study determined the sample size from the statistical data performed by Sailer and colleagues’ publication in 2007 (25) using G*power software version 3.1 (Heinrich Heine University, Düsseldorf, Germany) with a power of test = 0.90 and α error = 0.05 as shown in Equation 1, (Fig. 1).

*Which: Zβ = standard normal deviation = 1.28 (β error = 0.1), Zα = standard normal deviation = 1.96 (α error = 0.05), s = standard deviation (s1 = 2.3, s2 = 1.5), and µ1 - µ2 = mean difference between tested group = 0.8. The calculated sample size was employed 15 specimens per group.*

-Preparation of zirconia substructure specimens

One hundred twenty (120) disc specimens of 15 mm in diameter (Φ) and 1 mm thickness were prepared from the pre-shade-A2 partially-sintered 3Y-TZP (Bruxzir, Prismatik Dentalcraft, Hannover, Germany) block ( Table 1) using a diamond-coated disc at a velocity of 700 rounds per minute (rpm) by a water-cool sectioning machine (Isomet-1000, Buehler, Lake Bluff, IL, USA). The waterproof silicon carbide (SiC) abrasive sheets up to no. 5000 were used to grind the zirconia specimens in a wet condition with water using a grinding machine (Ecomet-3, Beuhler) at 50 rpm speed. The zirconia specimens were then sintered in a firing furnace (HiTherm, Hint-ELs, Griesheim, Germany) to obtain a final dimension of Φ 12 mm and thickness of 0.8 mm, owing to 20% shrinkage upon sintering. The zirconia specimens were unintentionally allocated for 8 groups (n = 15) for veneering with different ceramics.

-Preparation of veneering ceramics

Different types of the shade-A2 veneering ceramics comprising feldspathic- (Vitablocs; Vm, Vita-Zahnfabrik, Bad Sackingen, Germany), lithium disilicate-based glass- (IPS e.max-CAD; Em, Ivoclar-Vivadent, Schaan, Liechtenstein), zirconia-reinforced glass- (Vita-Suprinity; Vs, Vita-Zahnfabrik), and zirconia-reinforced lithium silicate- (Celtra-Duo; Cd, Dentsply, Hanau-Wolfgang, Germany) ceramic (Table 1) were prepared in disc-shape (n = 30/ceramic) through a low-velocity diamond saw (Isomet-1000, Buehler) and abraded with SiC abrasive paper up to grit no. 5000 using a water-cool grinding machine (Ecomet-3, Buehler) to obtain the definite dimension of Φ 12 mm and thickness 0.8 mm. Each kind of ceramics was inadvertently allocated into 2 subclasses (n = 15) consistent with CAD-bonded (Cb) or CAD-fused (Cf) to obtain the definite ceramic veneering zirconia comprising 0.8 mm zirconia substructure, 0.04 mm hybridized zone, and 0.8 mm veneering ceramic, determined by a digital caliper (Mitutoyo, Kawasaki, Japan).

CAD-bonded hybridized technique 

The surfaces of zirconia specimens to be hybridized with the Cb technique were blasted with 50 microns (µm) Al2O3 with 2.5 bar pressure in a blasted machine (Vario-basic, Renfert, Hilzingen, Germany) for 15 seconds (sec) by placing a blasting tip 10 mm distance and 45 degrees angulation from the specimen surface and ultrasonically cleaned in distilled water (Vitasonic-II, Vita-Zahnfabrik) for 15 minutes (min). Then, the hybridized surface of zirconia discs was applied with a zirconia-metal primer (Monobond-Plus, Ivoclar-Vivadent). The surface of veneering ceramics to be hybridized was etched with 5% HF acid (Ivoclar-Vivadent) for 20 sec, sprayed with distilled water, dehydrated in the air, applied with a zirconia-metal primer, coated with thin film resin adhesive (Variolink Esthetic, Ivoclar-Vivadent), then gently condensed against the zirconia specimen using a digital caliper (Mitutoyo) by controlling the resin cement thickness to be exactly 40 µm, and then polymerized with light curing unit (Mini-LED, Acteon, Norfolk, England) for 9 min.

-CAD-fused hybridized technique 

The surfaces of zirconia specimens to be hybridized with the Cf technique were prepared as previously described before being conjugated with the veneering ceramic discs. The powder-liquid creamy mixture of the fusion glass (e.max CAD Crystall-connect, Ivoclar-Vivadent) was gradually smeared on the whole conjugating side of the veneering ceramics and instantly condensed against the zirconia specimen using digital caliper (Mitutoyo) by controlling the layer of fusion glass to be precisely 40 µm, removed the surplus fusion glass with a micro-brush, and then sintered in the sintering furnace (Programat-P310, Ivoclar-Vivadent) according to the manufacturer’s sintering schedule.

-Determination of color parameters

**Figure 2:**
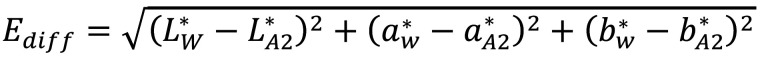
Equation 2.

The ColorQuest-XE spectrophotometer (Hunter, Reston, VA, USA) was utilized to determine the color parameters of ceramics veneered zirconia upon different hybridized techniques by setting at D65 illuminant, 100% UV, 10 degrees of observing angle, with a standard wavelength of 380–780 nm. An aperture of 4 mm in Φ was used to facilitate the precise spectrum directly on the specimen to eliminate the edge loss effect during measurement. To provide the analogous location for each sample during the measuring period, the clear repositioning jig was used to maintain the center of the specimen position. The determinations were made in CIELab (Commission Internationale de I’Eclairage). The L*, a*, and b* parameters were attained from the lightness, red-green, and yellow-blue coordinates, respectively on the white (W) (LW* = 96.70, aw* = 0.10, bw* = 0.20), and black (B) (LB* = 10.40, aB* = 0.40, bB* = 0.60) background. Then, the TP, CR, OP, and ∆Ediff were computed. The coordinates of the VITA classic shade-A2 (Vita Zahnfabrik) on a white background (LA2* = 65.61, aA2* = -0.50, bA2* = 5.54) were measured and used for determination the amount of color alteration through ∆Ediff values according to Equation 2 (20), (Fig. 2).

**Figure 3:**
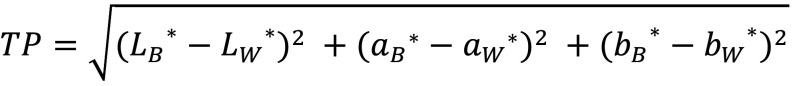
Equation 3.

The translucency was determined from the TP values that were calculated from the differences between color determinants on white (W) and black (B) backgrounds, according to Equation 3 (20), (Fig. 3).

**Figure 4:**
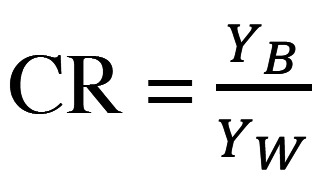
Equation 4.

**Figure 5:**
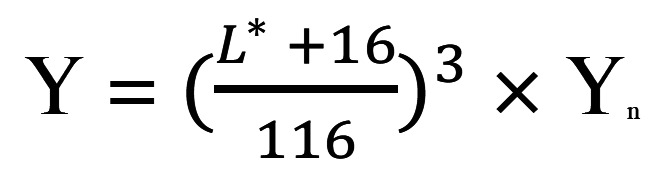
Equation 5.

The contrast was determined from the CR values according to Equations 4 and 5 in which the CR ranged from 0.00 (transparent) to 1.00 (perfectly opaque) (20). In terms of tristimulus color space, Y represents the brightness illuminance; YW and YB are the values of a sample placed on the white and black backgrounds, respectively; and Yn is equal to 100, (Figs. 4,5).

**Figure 6:**
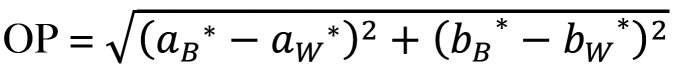
Equation 6.

The opalescence was determined from the OP values that were achieved by using Equation (6) (20), (Fig. 6).

-Determining the microstructure 

Three specimens from each group were ultrasonically cleaned in distilled water, dehydrated in the desiccator (Nokko, Nikko, Tokyo, Japan), and coated the surface with gold-palladium in the coating apparatus (K-500X, Emitech, Asford, England) using the current 10 mA for 3 min in the vacuum 130 Torr to evaluate the microstructure. The cross-sectional micrograph was also performed to determine the characteristics of the interface zone of ceramic veneered zirconia, the quality of zirconia-veneering hybridized surface, and the crystalline size of zirconia with scanning electron microscopy (SEM; S-3000N, Hitachi, Osaka, Japan). The Image-J software (U.S. National Institutes of Health, Bethesda, MD, USA) was utilized for measuring the grain size.

Determining the zirconia phase 

**Figure 7:**
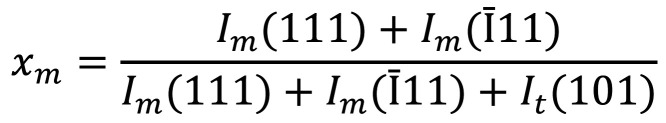
Equation 7.

**Figure 8:**
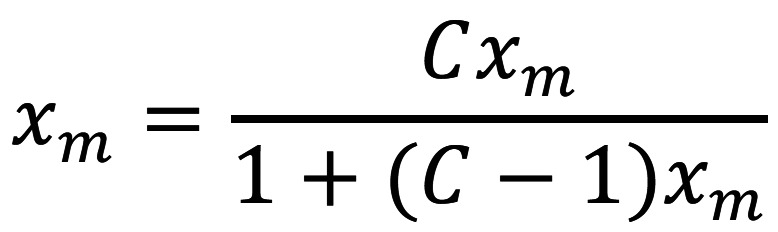
Equation 8.

**Figure 9:**
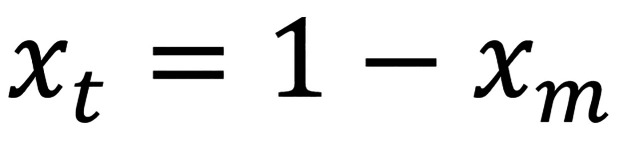
Equation 9.

The relative proportions of zirconia crystal phases were assessed with an X-ray diffractometer (XRD, MiniFlex-2, Rigaku, Tokyo, Japan). The specimens were probed with copper k-alpha (Cu Kα) emission at intervals of two seconds, with the angles of diffraction (2θ) ranging from 20 – 40 degrees (o) with 0.02o stepwise. The phases of zirconia were appraised by cross-reference with the Joint Committee of Powder Diffraction Standards database file (PDF). The analysis of the t- and m-phase proportion was performed by X’Pert-Plus software (Phillips, Almelo, Netherlands). The peaks for the m- and t- phases were identified with PDFs No. 37–1484 and 49–1642, correspondingly. The quantity of t-phase (Xt) and m-phase (Xm) to total crystalline phases was computed from the Garvie-Nicholson and Toraya formula as given in Equations 7, 8, 9 (26). The integrated intensities of the m-, and t-phases (It and Im) were estimated by matching the complementary peaks with a pseudo-Voigt distribution and considering the area beneath the curves. A correction factor (C) of 1.32 was established from a non-linear adjustment curve of the integrated intensity fractions versus volume fraction to take the impact of yttria doping on the lattice parameters into consideration, (Figs. 7,8,9)

-Statistical analysis

The data were accomplished for the normality test with the Shapiro-Wilk test, and the homoscedasticity test with Levene’s test using IBM SPSS V-28 statistics software (SPSS, Chicago, IL, USA). As the data revealed normal distribution and exhibited homoscedasticity (*p*>0.05), the two-way analysis of variance (ANOVA) along with Bonferroni multiple comparisons were implemented to detect substantial variations in the color parameters of different CAD/CAM ceramics veneered zirconia upon different hybridized techniques. A statistically significant difference was judged at *p*<0.05. Furthermore, descriptive statistics were employed to evaluate the optical properties, grain size, and relative phases of the zirconia.

## Results

**Figure 10:**
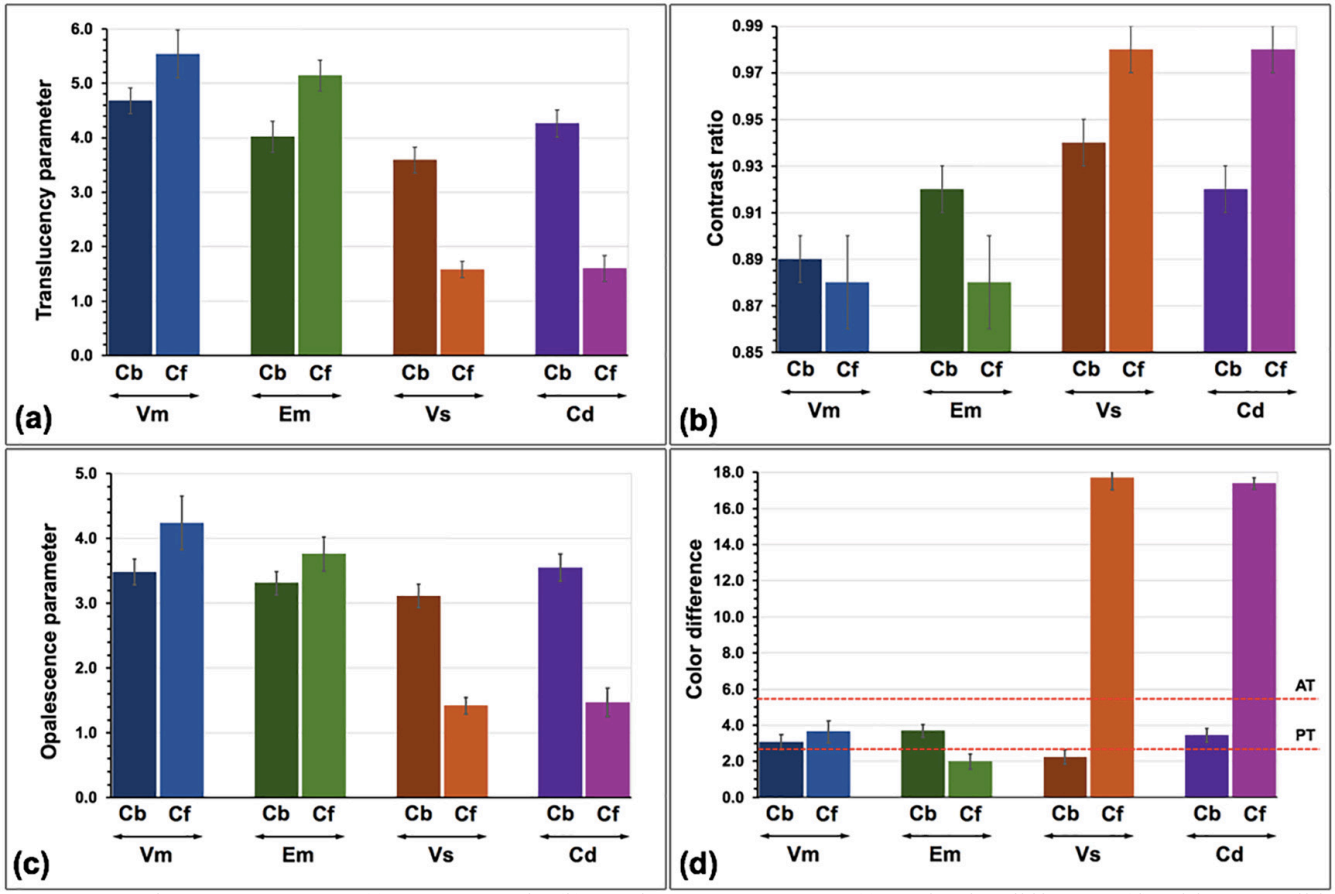
Translucency parameter (a), contrast ratio (b), opalescence parameter (c), and color difference (d) with perceptible threshold (PT) and acceptable threshold (AT) of Vitabloc (Vm), e.max CAD (Em), Vita Suprinity (Vs), Celtra Duo (Cd) ceramic veneered zirconia (Z) with either CAD-bonded (Cb) or CAD-fused (Cf) technique were shown.

**Figure 11:**
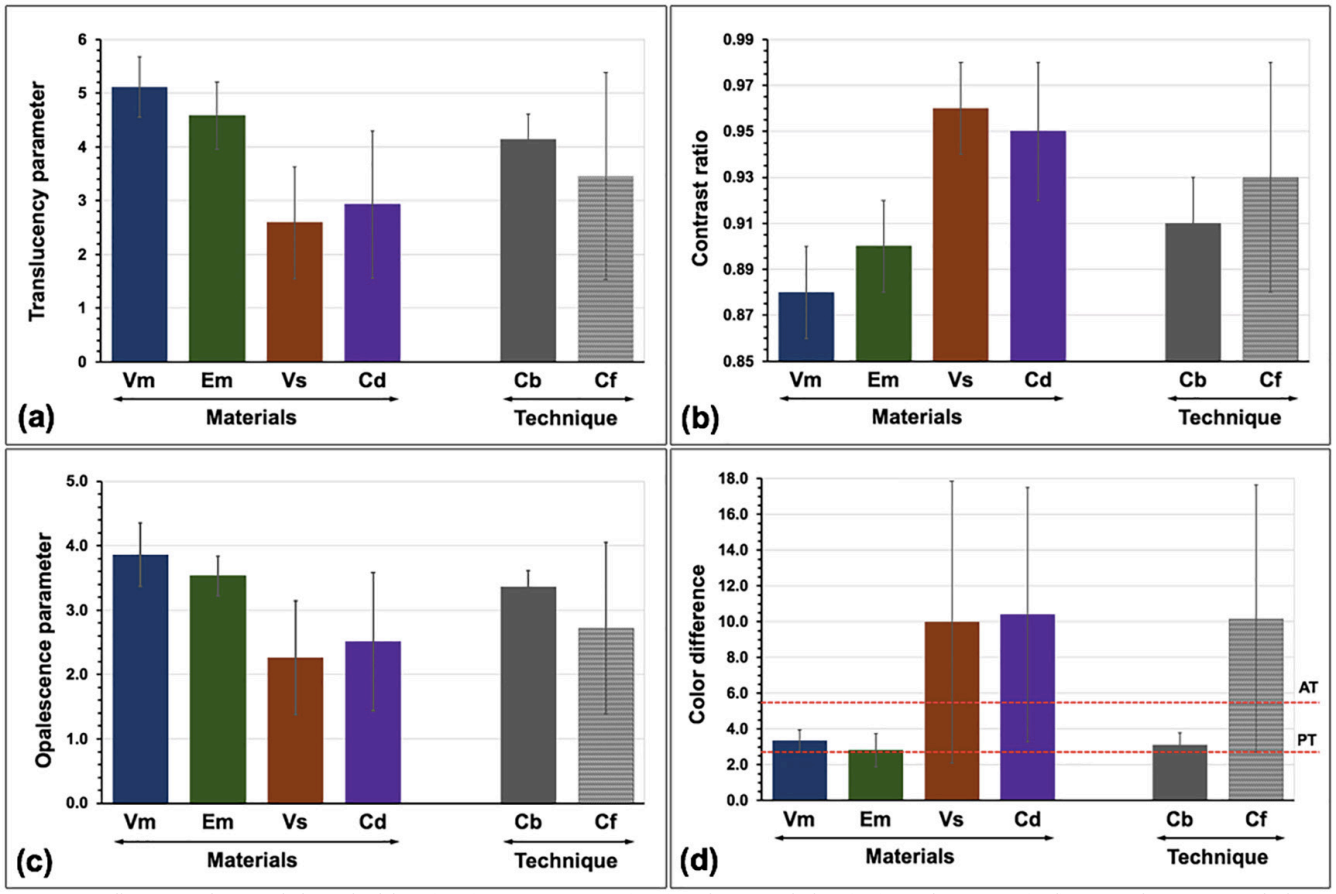
Influence of materials [Vitabloc (Vm), e.max CAD (Em), Vita Suprinity (Vs), Celtra Duo (Cd) ceramic] veneered zirconia with different hybridization techniques [CAD-bonded (Cb) or CAD-fused] (Cf) on translucency parameter (a), contrast ratio (b), opalescence parameter (c), and color difference (d) with perceptible (PT) and acceptable threshold (AT).

The mean of TP, CR, OP), and ΔEdiff, together with their standard deviation (SD) of experimental groups were presented (Fig. 10 and  Table 2). The VmCf group indicated the highest in both TP and OP values but the lowest in CR values compared to other groups. The VsCf group indicated the lowest in both TP and OP values but the highest in CR values compared to other groups. The highest ΔEdiff value was revealed in the VsCf group, while the lowest ΔEdiff value was revealed in the EmCf group, compared to others. ANOVA suggested a statistically significant difference in TP, CR, OP, and ΔEdiff owing to different veneering ceramics, hybridized techniques, and their interactions (*p*<0.05) ( Table 3). Post-hoc Bonferroni multiple comparison results for each color parameter were presented ( Table 4). Regarding the veneering ceramics, Bonferroni multiple comparisons indicated that different veneering ceramics possessed significant differences (*p*<0.05) in the TP, CR, OP, and ΔEdiff ( Table 4, Fig. 11). The Vm presented significantly higher TP and OP but lower CR than Em, Cd, and VS respectively (*p*<0.05). A similar ΔEdiff between Vs and Cd as well as between Vm and Em was demonstrated. Concerning the hybridized techniques, Bonferroni multiple comparisons demonstrated that different hybridized techniques created significant differences (*p*<0.05) in TP, CR, OP, and ΔEdiff ( Table 4, Fig. 11). The Cb presented significantly higher TP and OP, but lower CR and ΔEdiff than Cf (*p*<0.05). The Cb produced a more white, less red-yellow, and more green-blue color appearance in ceramic veneered zirconia than the Cf (Fig. 11). Concerning the interaction of veneering ceramics and hybridized techniques, Bonferroni multiple comparisons indicated that the interaction of hybridized techniques and veneering ceramics created a substantial difference in TP (*p*<0.05) except for EmCb-CdCb and VsCf-CdCf groups (*p*>0.05), together with substantial difference in CR (*p*<0.05) excepting VmCb-VmCf-EmCf, VmCf-EmCf, EmCb-CdCb, and VsCf-CdCf groups (*p*>0.05), and substantial difference in OP (*p*<0.05) excepting VmCb-EmCb-EmCf-CdCb, EmCb-VsCb-CdCb, EmCf-CdCb, and VsCf-CdCf groups (*p*>0.05), and additional substantial differences in ΔEdiff (*p*<0.05) excepting VmCb-CdCb, VmCf-EmCb-CdCb, EmCf-VsCb, and VsCf-CdCf groups (*p*>0.05) (Fig. 10,  Table 4). Concerning the color alteration compared to A2 VITA Classic shade (ΔEdiff), the EmCf and VsCb were considered within a PT (ΔEdiff ≤ 2.6), whereas the remaining groups except VsCf, and CdCf were considered within an AT (ΔEdiff ≤ 5.5). However, the impact of Vs and Cd veneering ceramics and Cf hybridized technique on the mean ΔEdiff values was beyond the AT.

**Figure 12:**
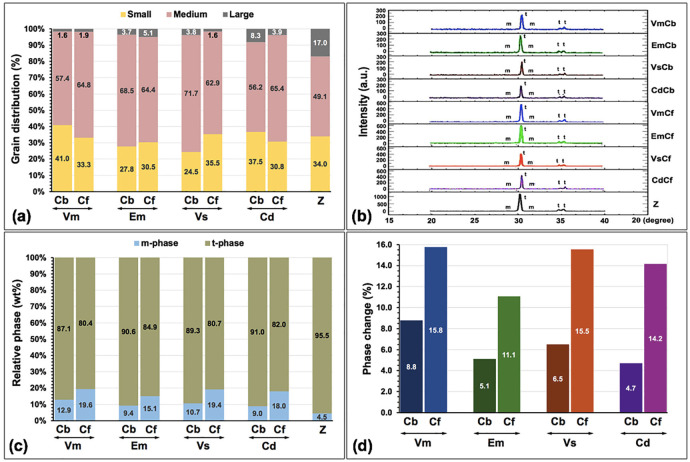
Grain distribution (a), phase intensity upon x-ray diffraction (b), relative phase of zirconia (c), and percentage of phase change (d) of Vitabloc (Vm), e.max CAD (Em), Vita Suprinity (Vs), Celtra Duo (Cd) ceramic veneered zirconia (Z) with either CAD-bonded (Cb) or CAD-fused (Cf) technique.

**Figure 13:**
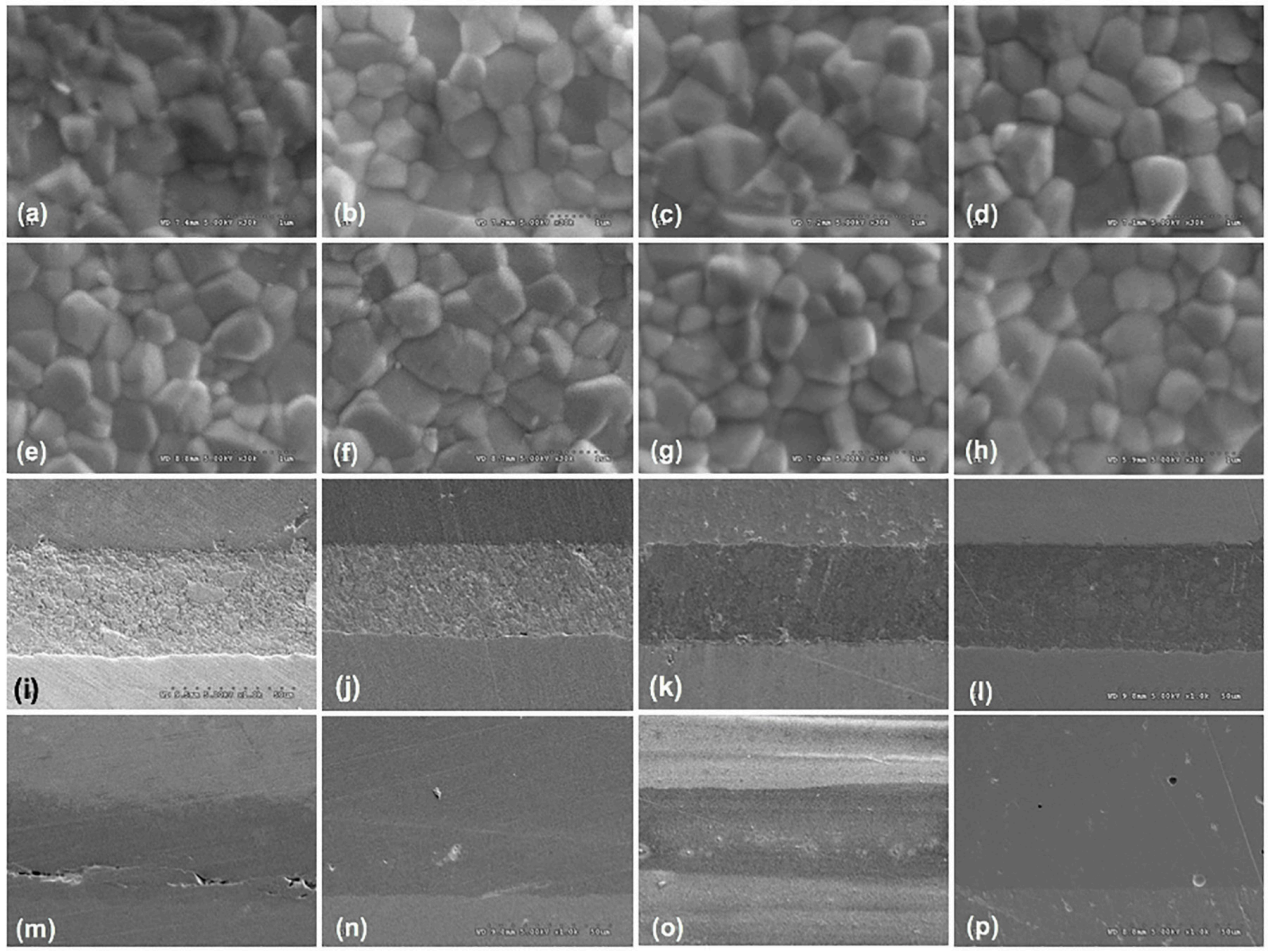
Scanning electron microscope photomicrographs of grain size and grain distribution of zirconia at ×30K magnification (a-h), and interfacial junction area at ×1000 magnification (i-p) of Vitabloc (Vm, a, e, i, m), e.max CAD (Em, b, f, j, n), Vita Suprinity (Vs, c, g, k, o), Celtra Duo (Cd, d, h, l, p) ceramic veneered zirconia with either CAD-bonded (Cb, a-d, i-l) or CAD-fused (Cf, e-h, m-p) technique.

The SEM micrographs displayed dissimilarity in the magnitude of zirconia crystalline particles upon varied veneering ceramics and hybridized techniques. The zirconia crystals were categorized into three groups related to their grain size: small (≤0.5 µm), medium (0.5<x≤0.7 µm), and large (>0.7 µm), and their relative percentage of grain distribution was presented (Fig. 12a, 13,  Table 2). The relative percentage of the grain size distribution for the 3Y-TZP substructure was affected by the veneering ceramics and hybridized techniques (Fig. 12a and  Table 2). The microstructures of the zirconia substructure and the zirconia-veneer interface for all investigated groups were shown (Fig. 13 (a-p)). From the cross-sectional core-veneer ceramics, All interfacial junctions among veneering ceramic/resin cement/zirconia substructure were well distinguished (Fig. 13 (i-l)) while the interfacial junction among veneering ceramic/fusion glass/zirconia substructure were well incorporated (Fig. 13 (m-p)). The harmonized conjugations were noticeable for all interfaces (Fig. 13). The quantity of the zirconia phase was XRD-analyzed as illustrated that the spectral positions of the crystalline phase synchronized with the associating t- and m-phases of zirconia within the firmness of the data (Fig. 12 (b,c),  Table 2). The pattern of XRD exhibited a large quantity of t-phase and a tiny quantity of m-phase. The t-phase was identified at the 2θ degree of 30.11°, 34.53°, and 35.09°. The m-phases were detected at 27.79° and 31.12°. The XRD data matched the crystallographic patterns signified by the PDF standard. The proportional intensities (wt.%) of m-phases compared to the total quantity of zirconia phases disclosed the alterations in the quantity of the phase conversion from the t→m phase, owing to the influence of veneering ceramics and hybridized techniques ( Table 2, Fig. 12 (b,c)). The quantity of phase content was associated with the influence of ceramic veneering materials and hybridized techniques. An increase in the relative quantity of t→m phase transformation was greater for CAD-fused hybridized than CAD-bonded hybridized technique (Fig. 12d,  Table 2).

## Discussion

To accomplish natural color characteristics restorations, superior translucency, enriched opalescence, and minimized color alteration of ceramic veneered zirconia, different types of ceramic materials veneering zirconia substructures either by CAD-fused or CAD-bonded hybridized techniques were examined in the present study. The substantial statistics differences for whole color parameters including TP, CR, OP, and ΔEdiff of varied veneering ceramics, with different hybridized techniques, and their interactions were discovered. Hence, all null hypotheses were rejected.

Translucency is a fundamental characteristic of replicating the natural tooth appearance specifically in the esthetic zone, which is defined by TP and CR values of ceramic veneering zirconia (9,18). This current study indicated substantial impacts on the TP of ceramic veneer zirconia amongst each ceramic material. The Vm is extremely impacted on TP ceramic veneered zirconia, trailed by Em, Cd, and Vs. Contrariwise, Vs supremely influenced on CR of ceramic veneered zirconia, trailed by Cd, Em, and Vm. The study inferred that the feldspathic ceramic provided better translucence to ceramic veneered zirconia than the LS2 glass ceramic, ZLS ceramic, and zirconia-reinforced glass ceramic, respectively. Translucency of ceramics can be affected by the thickness, and crystal microstructure including crystal volume, refractive index, particle size, and number of firing cycles (6,17). In the present study, TP was altered on account of different veneering ceramics and hybridized techniques. This is possibly related to the varied crystalline contents of each veneering ceramic as well as the reflectance at the interface between the zirconia substructure and veneering ceramics (19). Concerning the hybridized techniques, the Cf technique needs additional firing to fuse veneering ceramics to the zirconia substructure, while Cb does not. For the Vm and Em groups, the Cf technique produced higher TP than the Cb technique. Despite the Vs and Cd groups, the Cf technique produced lower TP than the Cb technique. The results confirmed a powerful association between Cr and TP; as CR decreases, TP increases, which was supported by other studies (18,27). Conversely, a weak association between direct transmittance and CR was found for CAD/CAM ceramics (27). It was presumed that CR values, measured from diffuse reflectance, are not capable of detection upon minute alterations in light transmission as the materials possess extreme scattering and absorption coefficients (27). The CR was suggested only for ceramics occupied for at least 50% of total transmission (18). As for CR values in the present study, the Cf technique showed lower CR than the Cb technique in the Vm and Em groups, while the Cf technique presented superior CR than the Cb technique in the Vs and cd groups. These results confirmed that veneering ceramics, hybridized techniques, and their interaction significantly affected the TP and CR of the specimens.

Concerning microstructure, the optical characteristics of ceramic material were influenced by the nature, shape, relative quantity, and distribution of particle size of the crystalline phases and porosity. The Cf technique showed a higher percentage of t→m phase transformation than the Cb technique, this could account for the varied characterization of color in the Cf technique. As for TP values, the highest TP value went to VmCf and the lowest TP value also went to CdCf. As well as for CR values, the highest CR value went to CdCf and the lowest CR value went to VmCf. In the Cb technique, both TP and CR values did not change as much as in the Cf technique. Larger crystal content produces superior fracture strength and, in contrast, can reduce translucence (5). The Vs is a feldspathic ceramic reinforced with sanidine, containing a crystal substance of approximately 30%. The Em is a LS2 glass ceramic, containing a crystal substance of approximately 65%. This confirmed that crystal substance motivates the optical characteristics of ceramics as supported by another study (18). Both Vs and Cd are ZLS ceramics, comprising 10% of zirconia suspended in the lithium silicate glass matrix resulting in 4 times smaller silicate crystals, meaning a higher amount of glass substance and superior translucence than classical LS2 ceramics. The Cd attained higher direct transmittance values than Em (2). Nevertheless, the newest study mentioned that no definite correlation between translucency and contrast since these properties tend to be material-specific (2). Furthermore, the close matching of the refractive index of the crystalline structure and glass matrix is also essential in regulating the translucency and intrinsic appearance of ceramics (18,28). Previous investigations reported that glass-ceramics have an inferior refractive index of 1.5, while zirconia possesses a superior refractive index of 2.2. Less crystal substance coupled with a close refractive index of crystal structure to that of the glass matrix instigates less light scattering (28). This might be the explanation for the highest TP values of Vm upon veneering zirconia (18). Concerning TP of ceramic material per se used in this study, the highest TP was occupied in Cd (17.23±0.55), followed by Vm (15.39±0.89), Vs (14.61±0.88), Em (14.47±0.75), and Zirconia (6.25.47±40), respectively. However, when veneering ceramics bonded to zirconia with resin cement (TP = 41.39±0.51), the VmCb has the highest TP followed by CdCb, EmCb, and VsCb, respectively. In the CAD-fused technique, the order from the highest to lowest TP were VmCf, EmCf, CdCf, and VsCf, respectively. The crystalline volume and refractive index in the present study likewise vary from those of other ceramic systems. Vs and Cd have high crystalline contents; however, the manufacturers claim that the crystalline structure is fine causing these systems to be more translucent. These factors might also have affected the results of the present study.

Opalescence is generated by the scattering effect of the tiny wavelengths of the spectrum of visible light on the small particle sizes of ceramic material, giving the ceramic a bluish appearance in the reflective light and an orange-brown appearance in the transmitted light. To fabricate extremely esthetic ceramic restorations that imitate natural tooth appearance, the ceramics that are capable of generating opalescence ought to be utilized. The OP values of 3.01-7.64 of 1.0 mm thickness of glass ceramics were reported (18), which were greater than the veneering ceramics in this study. Ceramics with superior OP were related to the rising quantities of certain oxides, for example, Y2O3, ZrO2, SnO2, and V2O5 (16). Previous study reported that the OP of core material, veneering ceramics, A2-shade ceramic veneered cores were 1.6 - 6.1, 2.0 - 7.1, and 1.3 - 5.0, respectively, which indicated significant influence of types of material on opalescence (29). This study denoted that the OP values of the A2-ceramic veneered zirconia ranged from 1.42 - 4.24, which were in the same range as the previous study (29). Opalescence is associated with translucence, in which the low translucency of the zirconia substructure probably influences the OP value of ceramic veneered zirconia in this study. Concerning hybridized techniques, the Cf technique showed higher OP for Vm and Em veneered zirconia than the Cb technique. Conversely, the Cf technique showed lower OP for Vs and Cd veneered zirconia than the Cb technique. The study confirmed that hybridized techniques, veneering ceramics, and their interaction significantly affected the OP of the specimens. As there was no standard guidance to identify opalescence, the decision concerning the materials being considered as opalescence could not be justified (21). Nevertheless, the OP values in this study were lower than the OP of human enamel (22.9±1.9), thus the ceramics examined in this study probably be classified as non-opalescence.

Regarding color appearance difference between the two subjects is represented by the arithmetic distance difference between L*a*b* coordinates of two materials (∆Ediff), which may not be detectable by the human eye. The level of visual perception or clinically acceptable color appearance differences varies and is based on an individual basis. However, it was described that ∆Ediff value as “clinical imperceptible” (∆Ediff < 2.6), “clinical acceptable” (∆Ediff = 2.6 – 5.5), and “clinical unacceptable” (∆Ediff > 5.5) appear to be coherent with the non-expert consideration in clinical practice, which normally related to the patient’s concern (23,24). It is evident that no matter how the veneering technique is implemented, the b* value significantly increases once veneering, indicating the tendency of ceramic veneered zirconia to develop a more yellow appearance. Veneering ceramics materials and hybridized techniques tested in this study tended to demonstrate individual color appearance. This investigation is essential for dentists as well as dental technicians, in that they should be careful when selecting the veneering ceramics to be used with each hybridized technique to the zirconia; the color change of some groups was higher than the clinically acceptable level (∆Ediff > 5.5). Very little data is available on the shade reproduction with different veneering ceramics for zirconia substructure. The differences between the ceramic veneered zirconia substructure and the ceramic veneered metal substructures are mainly related to the difference in the coefficient of thermal expansion. It is hence reasonable to conclude that most of the factors influencing the color appearance of the ceramic veneering metal may affect the ceramic veneering zirconia as well (1,11). In the present study, regardless of the hybridized techniques, color changes occurred after combining veneering ceramic with zirconia substructure. This study also confirmed that the difference in hybridized technique caused a substantial effect on the color appearance of ceramic veneered zirconia restoration, whilst the ∆Ediff changed. Previous studies stated that the veneering ceramic color was influenced by the ceramic thickness, underlying substrate, and the interaction between them, which supported the result of this study (16,30). This study confirmed that ceramics veneering zirconia, with the same shade, but from different ceramic types exhibited differences in color appearance, which was consistent with the previous studies (30). The CIELab coordinates for Vita classical shade (VITA Zahnfabrik) show L*, a*, and b* values for A2 shade equal to 74.0, 1.7, and 19.3 respectively. Compared to the results obtained from this study, the Vita classic shade guide was redder and yellower than the color appearance of the entire experimental groups. The result indicated that upon the use of the claimed shade guide in the process of shade selection for the restoration, the outcome of the shade of the restoration is certainly not the same as expected. Thus, the clinician should keep in mind that the final shade of the restoration is almost always different from the selected shade, due to the different materials used to fabricate restoration and the technique in combination, as discovered in this study.

## Conclusions

The study herein revealed that types of veneering ceramic, hybridized technique, and their interaction affected the color characteristics of ceramic veneered zirconia. Veneering zirconia with either Vm or Em provided greater translucency and opalescence, but less contrast and color alteration than veneered with Vs or Cd. Cf hybridized technique yielded less translucency and opalescence, but higher contrast and color alteration to ceramic veneered zirconia than Cb hybridized technique. Zirconia veneering with either Vm or Em and with either Cb or Cf hybridized technique, appeared to produce better translucence and opalescence, with less contrast and color alteration than veneering with either Vs or Cd and with either Cb or Cf hybridized technique. However, the color alteration for different ceramics veneering zirconia, with different hybridized techniques remained within an accepTable limit, except for both Vs and Cd upon the Cf hybridized technique.

-Clinical implications

To produce CAD-CAM ceramic veneered zirconia restoration with enhanced translucency, opalescence, and optimal contrast and color alteration, it is recommended to veneer the zirconia substructure with either feldspathic or lithium disilicate-based glass ceramic compared to zirconia reinforced glass ceramic and zirconia reinforced lithium silicate ceramic. Ceramic veneering zirconia with a CAD-bonded hybridized technique produces better translucency, opalescence, and less contrast and color alteration than veneering with a CAD-fused hybridized technique. Nevertheless, it could be deemed accepTable for color alteration, meaning accepTable color stability of zirconia to be veneered with any veneering ceramic through either CAD-fused or CAD-bonded technique, except only zirconia-reinforced glass ceramic and zirconia reinforced lithium silicate ceramic need to be veneered by CAD-bonded hybridized technique.

## Figures and Tables

**Table 1:** Material, brand, abbreviation (Abv.), manufacturers, batch number, Young’s modulus, coefficient of thermal expansion (CTE, X10-6 /K) of materials used in this study.

Material	Brand	Abv.	Manufacturer	Batch no.	Young's modulus	CTE
Yttria-stabilized tetragonal zirconia polycrystalline	Bruxzir	Z	Prismatik Dentalcraft, Hannover, Germany	B1379641	210	11
Feldspathic ceramic	Vitablocs	Vm	Vita-Zahnfabrik, Bad Sackingen, Germany	49470	65	9.4±1
Lithium disilicate-based glass ceramic	IPS e.max-CAD	Em	Ivoclar-Vivadent, Schaan, Leichtenstein	V43832	95	10.1±0.5
Zirconia-reinforced glass ceramic	Vita-Suprinity	Vs	Vita-Zahnfabrik, Bad Sackingen, Germany	66418	70	12.3
Zirconia-reinforced lithium silicate ceramic	Celtra Duo	Cd	Dentsply, Hanau-Wolfgang, Germany	18028463	70	11.8

**Table 2:** Mean, standard deviation (SD) of translucency parameter (TP), contrast ratio (CR), opalescence parameter (OP), color difference (∆Ediff), relative monoclinic (m-), and tetragonal (t-) phase content (wt.%), percentage of phase change (%) and percentage of small (s), medium (m), and large (l) grain size distribution (%) of Vitabloc (Vm), e.max CAD (Em), Vita Suprinity (Vs), Celtra Duo (Cd) ceramic veneered zirconia (Z) with either CAD-bonded (Cb) or CAD-fused (Cf) technique.

Group	n	TP	CR	OP	∆E_diff_	Phase (wt%)		Phase change (%)	Grain size (%)
		Mean±SD	Mean±SD	Mean±SD	Mean±SD	m-	t-	t → m	s / m / l
VmCb	15	4.68±0.24	0.89±0.01	3.48±0.20	3.07±0.42	12.9	87.1	8.8	41.0 / 57.4 / 1.6
VmCf	15	5.54±0.44	0.88±0.02	4.24±0.41	3.64±0.62	19.6	80.4	15.6	33.3 / 64.8 / 1.9
EmCb	15	4.02±0.28	0.92±0.01	3.31±0.18	3.68±0.34	9.4	90.6	5.1	27.8 / 68.5 / 3.7
EmCf	15	5.14±0.28	0.88±0.02	3.76±0.26	1.97±0.42	15.1	84.9	11.1	30.5 / 64.4/ 5.1
VsCb	15	3.59±0.24	0.94±0.01	3.11±0.18	2.24±0.39	10.7	89.3	6.5	24.5 / 71.7 / 3.8
VsCf	15	1.58±0.15	0.98±0.01	1.42±0.13	17.69±0.66	19.4	80.7	15.5	35.5 / 62.9 / 1.6
CdCb	15	4.26±0.25	0.92±0.01	3.55±0.21	3.44±0.38	9	91	4.7	37.5 / 56.2 / 8.3
CdCf	15	1.60±0.24	0.98±0.01	1.47±0.22	17.37±0.31	18	82	14.2	30.8 / 65.4 / 3.9
Z	15	-	-	-	-	4.5	95.5	-	34.0 / 49.1 / 17.0

**Table 3:** Two-way ANOVA of (a) translucency parameter (TP), (b) contrast ratio (CR), (c) opalescence parameter (OP), (d) color difference (∆Ediff) of CAD-CAM ceramic veneered zirconia with either CAD-bonded or CAD-fused technique.

(a) ANOVA of TP upon different factors					
Source	SS	df	MS	F	p
Corrected Model	234.785	7	33.541	435.026	.001
Intercept	1734.063	1	1734.063	22490.984	.001
Ceramic	136.667	3	45.556	590.863	.001
Technique	13.637	1	13.637	176.879	.001
Material * Technique	84.480	3	28.160	365.237	.001
Error	8.635	112	.077		
(b) ANOVA of CR upon different factors					
Corrected Model	.192	7	.027	178.927	.001
Intercept	101.970	1	101.970	666789.191	.001
Ceramic	.133	3	.044	289.837	.001
Technique	.006	1	.006	41.810	.001
Material * Technique	.052	3	.017	113.722	.001
Error	.017	112	.000		
(c) ANOVA of OP upon different factors					
Corrected Model	114.011	7	16.287	294.593	.001
Intercept	1109.990	1	1109.990	20076.760	.001
Ceramic	54.153	3	18.051	326.493	.001
Technique	12.427	1	12.427	224.763	.001
Material * Technique	47.432	3	15.811	285.970	.001
Error	6.192	112	.055		
(d) ANOVA ∆E_diff_ upon different factors					
Corrected Model	4790.565	7	684.366	3225.173	.001
Intercept	5289.511	1	5289.511	24927.567	.001
Ceramic	1518.467	3	506.156	2385.330	.001
Technique	1496.248	1	1496.248	7051.281	.001
Material * Technique	1775.849	3	591.950	2789.647	.001
Error	23.766	112	.212		

NB: SS: sum of squares, df: degree of freedom, MS: mean square, F: F-ratio.

**Table 4:** Post hoc Bonferroni multiple comparisons of (a) translucency parameter (TP), (b) contrast ratio (CR), (c) opalescence parameter (OP), (d) color difference (∆Ediff) of different ceramic (C) including Vitabloc (Vm), e.max CAD (Em), Vita Suprinity (Vs), Celtra Duo (Cd) ceramic veneered zirconia (Z) with either CAD-bonded (Cb) or CAD-fused (Cf) technique (T).

(a) Post hoc of TP as a function of ceramic, technique, and ceramic*technique interaction															
C	Vm	Em	Vs		Cd		C*T	VmCb	VmCf	EmCb	EmCf	VsCb	VsCf	CdCb	CdCf
Vm	1	.001	.001		.001		VmCb	1	.001	.001	.001	.001	.001	.001	.001
Em		1	.001		.001		VmCf		1	.001	.004	.001	.001	.001	.001
Vs			.000		.001		EmCb			1	.001	.002	.001	.600	.001
Cd					1		EmCf				1	.001	.001	.000	.001
							VsCb					1	.001	.001	.001
T	Cb	Cf					VsCf						1	.001	1
Cb	1	.001					CdCb							1	.001
Cf		.001					CdCf								1
(b) Post hoc of CR as a function of ceramic, technique, and ceramic*technique interaction															
C	Vm	Em	Vs		Cd		C*T	VmCb	VmCf	EmCb	EmCf	VsCb	VsCf	CdCb	CdCf
Vm	1	.001	.001		.001		VmCb	1	.150	.001	1	.001	.001	.001	.001
Em		1	.001		.001		VmCf		1	.001	1	.001	.001	.001	.001
Vs			1		.007		EmCb			1	.001	.001	.001	1	.001
Cd					1		EmCf				1	.001	.001	.001	.001
							VsCb					1	.001	.001	.001
T	Cb	Cf					VsCf						1	.001	1
Cb	1	.001					CdCb							1	.001
Cf		1					CdCf								1
(c) Post hoc of OP as a function of ceramic, technique, and ceramic*technique interaction															
C	Vm	Em	Vs		Cd		C*T	VmCb	VmCf	EmCb	EmCf	VsCb	VsCf	CdCb	CdCf
Vm	1	.001	.001		.001		VmCb	1	.001	1	.058	.001	.001	1	.001
Em		1	.001		.001		VmCf		1	.001	.001	.001	.001	.001	.001
Vs			1		.001		EmCb			1	.001	.679	.001	.176	.001
Cd					1		EmCf				1	.001	.001	.484	.001
							VsCb					1	.001	.001	.001
T	Cb	Cf					VsCf						1	.001	1
Cb	1	.001					CdCb							1	.001
Cf		1					CdCf								1
(d) Post hoc of ∆E_diff_ as a function of ceramic, technique, and ceramic*technique interaction															
C	Vm	Em	Vs		Cd		C*T	VmCb	VmCf	EmCb	EmCf	VsCb	VsCf	CdCb	CdCf
Vm	1	.001	.001		.001		VmCb	1	.028	.014	.001	.001	.001	.946	.001
Em		1	.001		.001		VmCf		1	1	.001	.001	.001	1	.001
Vs			1		.003		EmCb			1	.001	.001	.001	1	.001
Cd					1		EmCf				1	1	.001	.001	.001
							VsCb					1	.001	.001	.001
T	Cb	Cf					VsCf						1	.001	1
Cb	1	.001					CdCb							1	.001
Cf		1					CdCf								1

## Data Availability

The datasets used and/or analyzed during the current study are available from the corresponding author.
